# Interkingdom horizontal gene transfer in plants: a perspective on methodological limitations and evolutionary alternatives

**DOI:** 10.3389/fpls.2026.1789570

**Published:** 2026-03-20

**Authors:** Vinicio Armijos-Jaramillo, Kevin Aguirre-Carvajal

**Affiliations:** 1 Bio-Cheminformatics Research Group, Universidad de Las Américas, Quito, Ecuador; 2 Carrera de Ingeniería en Biotecnología, Facultad de Ingeniería y Ciencias Aplicadas, Universidad de Las Américas, Quito, Ecuador; 3 Computer Science Faculty, University of A Coruna, CITIC-Research Center of Information and Communication Technologies, A Coruña, Spain

**Keywords:** alternative evolutionary explanations, eukaryotes, interkingdom horizontal gene transfer, methodological limitations, plant genome evolution

## Abstract

Over the past decade, numerous studies have suggested that plant genomes have been substantially influenced by interkingdom horizontal gene transfer (iHGT). Although the prevalence of this process in eukaryotes—particularly in multicellular organisms—remains an active area of discussion, many reported plant iHGT candidates have not always been examined in light of alternative evolutionary explanations. This raises the possibility that the contribution of iHGT to plant genome evolution may be less pervasive than currently proposed. In this perspective article, we revisit the evidence commonly used to support iHGT in plants and consider plausible alternative scenarios that could generate similar phylogenetic patterns. We also outline key limitations of the methods currently used to detect iHGT and suggest directions for improving future analyses. Our goal is to encourage careful evaluation of the criteria applied to infer iHGT and to promote a balanced view of its potential impact on plant genome evolution.

## Introduction

Horizontal gene transfer (HGT) is a well-established adaptive mechanism in bacteria; however, its significance in eukaryotes—particularly in cases involving distantly related organisms, such as interkingdom HGT (iHGT)—remains a topic of active debate (Ku and Martin, 2016; Martin, 2017, 2018; Boto, 2018; Leger et al., 2018; Roger, 2018; Aguirre-Carvajal et al., 2024, 2025; Bremer et al., 2025). Several studies claim that iHGT has played a relevant role in plant evolution, suggesting that it shaped key adaptive transitions, for example during the colonization of land and the early evolution of streptophytes (Ma et al., 2022). Other authors propose that iHGT has had a substantial impact on the development of specific traits, including abiotic stress resistance (Li et al., 2022), carbohydrate metabolism (Haimlich et al., 2024), secondary metabolism and biodegradation (Wu et al., 2024), as well as growth and development (Ma et al., 2022).

Consistent with these interpretations, numerous studies in recent years have reported sets of individual genes putatively transferred from other kingdoms—mainly bacteria—to plant nuclear genomes, implying that this process is common in plant evolution. For instance, Cai et al. (2021) reported 93 HGT events; Li et al. (2022) identified 235 plant genes associated with abiotic stress with a putative iHGT origin; Ma et al. (2022) reported 593 events; Haimlich et al. (2024) described 58 unique genes transferred from bacteria to plants; and Wu et al. (2024) reported 322 iHGT events. Taken together, these publications report 1,301 putative iHGT events in plant genomes within just the last five years. Although some of these cases may represent genuine transfers, the sheer number of claims calls for careful and critical evaluation.

According to the arguments presented in these studies, iHGT has occurred repeatedly throughout plant evolution, providing key tools for adaptation to new environments and for the diversification of metabolic pathways. This reasoning implies that hundreds of genes have entered plant genomes, either conferring novel functions, modifying existing ones, or initially becoming fixed through genetic drift before acquiring adaptive relevance in newly evolving species. If this panorama is correct, iHGT would represent a major—perhaps essential—evolutionary force in plants. However, this raises an important question: what if the evidence supporting iHGT has been misinterpreted? Could alternative evolutionary processes account for the same patterns? In particular, could the absence of detectable eukaryotic homologs in current databases bias phylogenetic inferences and lead to erroneous conclusions of iHGT?

In this article, we revisit the evidence currently available for iHGT in plants and re-evaluate alternative explanations for the same observations. We also discuss key limitations of existing HGT detection approaches and outline potential strategies to improve the identification of iHGT events in plant genomes.

## The “last-one-out” explanation

In recent years, the increasing number of reported HGT cases has been accompanied by a tendency to interpret highly discordant gene tree topologies as evidence of gene transfer (Yue et al., 2012; Cote-L’Heureux et al., 2022; Ma et al., 2022; Haimlich et al., 2024). At the same time, some of these unexpected patterns may also be explained by alternative evolutionary scenarios that, in certain cases, require fewer assumptions. We therefore suggest that these possibilities be carefully evaluated alongside HGT hypotheses when interpreting discordant trees.

**Figure 1:**
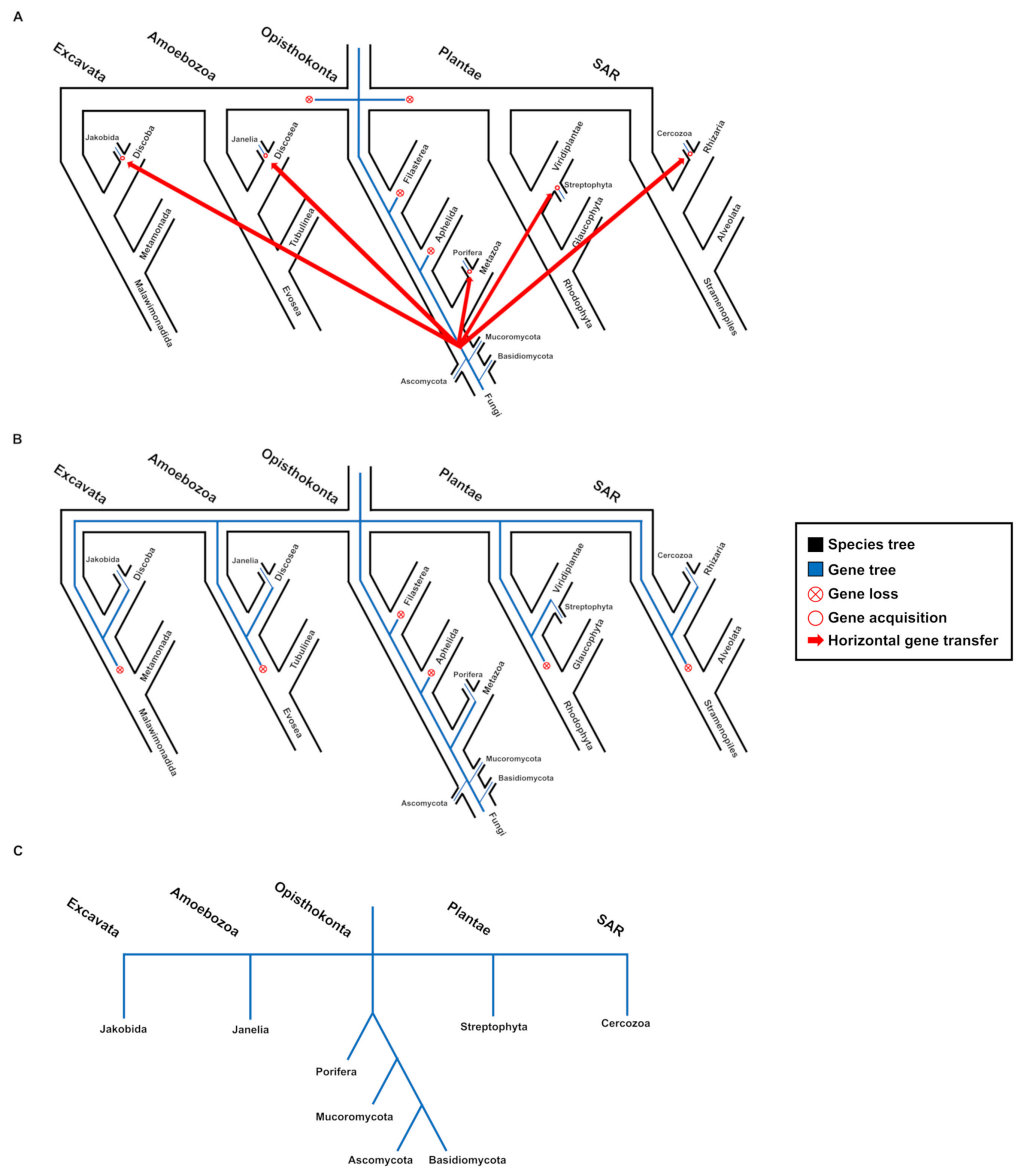
Schematic representation of two alternative evolutionary scenarios explaining an anomalous phylogeny characterized by numerous homologs in fungi and only a few sequences in other eukaryotic groups. **(A)** Interkingdom HGT scenario: this interpretation requires assuming five independent horizontal transfers of the same fungal gene into distinct eukaryotic lineages, along with multiple subsequent gene losses. **(B)** Differential gene loss scenario (“last-one-out”): here, the pattern can be explained by at least six independent gene losses across several eukaryotic lineages, without invoking repeated HGT. **(C)** Gene tree topology reconstructed with currently available data.

The recent study by Bremer et al. (2025) introduced a model to quantify the likelihood of “last-one-out” patterns— or clades where the majority of species have lost a specific gene of interest, leaving it retained in only one or few descendants. Such patterns can easily be misinterpreted as evidence of HGT and may account for many cases reported in earlier studies. For a gene to be genuinely acquired through HGT, it must overcome substantial biological barriers (Keeling and Palmer, 2008; Fitzpatrick, 2011), become fixed in a recipient population—a process difficult to reconcile with multiple independent acquisitions—and persist over long evolutionary timescales under changing selective regimes. By contrast, a last-one-out scenario requires only differential gene loss to produce the same phylogenetic topology. An analogous outcome could also result from ancient incomplete lineage sorting early in the history of the gene. **Figure 1** illustrates how both iHGT and lineage-specific gene loss can lead to effectively indistinguishable tree structures.

For example, the phylogeny of XM_024681535.1 reported by (Wu et al., 2024) includes homologs in fungi, Lycopodiopsida, Klebsormidiophyceae, Chlorophyta, Metazoa, and Oomycetes. Interpreting this topology as evidence of HGT would require assuming that a fungal donor independently transferred the same gene to multiple eukaryotic lineages—an unlikely scenario. In contrast, the “last-one-out” hypothesis invokes differential gene loss across most groups, with preferential retention in fungi, to explain the same pattern. Comparable topologies are observed in other candidates from the same study, such as XM_002971401.2 and AagrBONN_evm.model.Sc2ySwM_344.1584, and are consistent with previous work emphasizing gene loss as a major force shaping eukaryotic genomes (Domazet-Lošo et al., 2024). Similar patterns are also evident in Klebsormidium_nitens-lunzao_knT010613.1 from Ma et al. (2022), XP_002952597.1 from Li et al. (2022), and KAA6430084 from Puginier et al. (2024). Although some cases may indeed reflect genuine transfers, these examples highlight the need to consider alternative evolutionary processes—particularly differential gene loss and incomplete lineage sorting—before attributing anomalous phylogenies to iHGT.

## The lack of available homologs

Another plausible explanation for phylogenetic patterns interpreted as HGT is incomplete representation of homologs in public databases. As shown by Aguirre-Carvajal et al. (2024) in fungi, limited taxonomic sampling substantially increases the rate of false-positive HGT detections. When homologs are absent from eukaryotic datasets—either because the relevant lineages have not yet been sequenced or because the genes were lost in some branches—phylogenetic reconstructions can misleadingly favor an iHGT interpretation.

**Figure 2:**
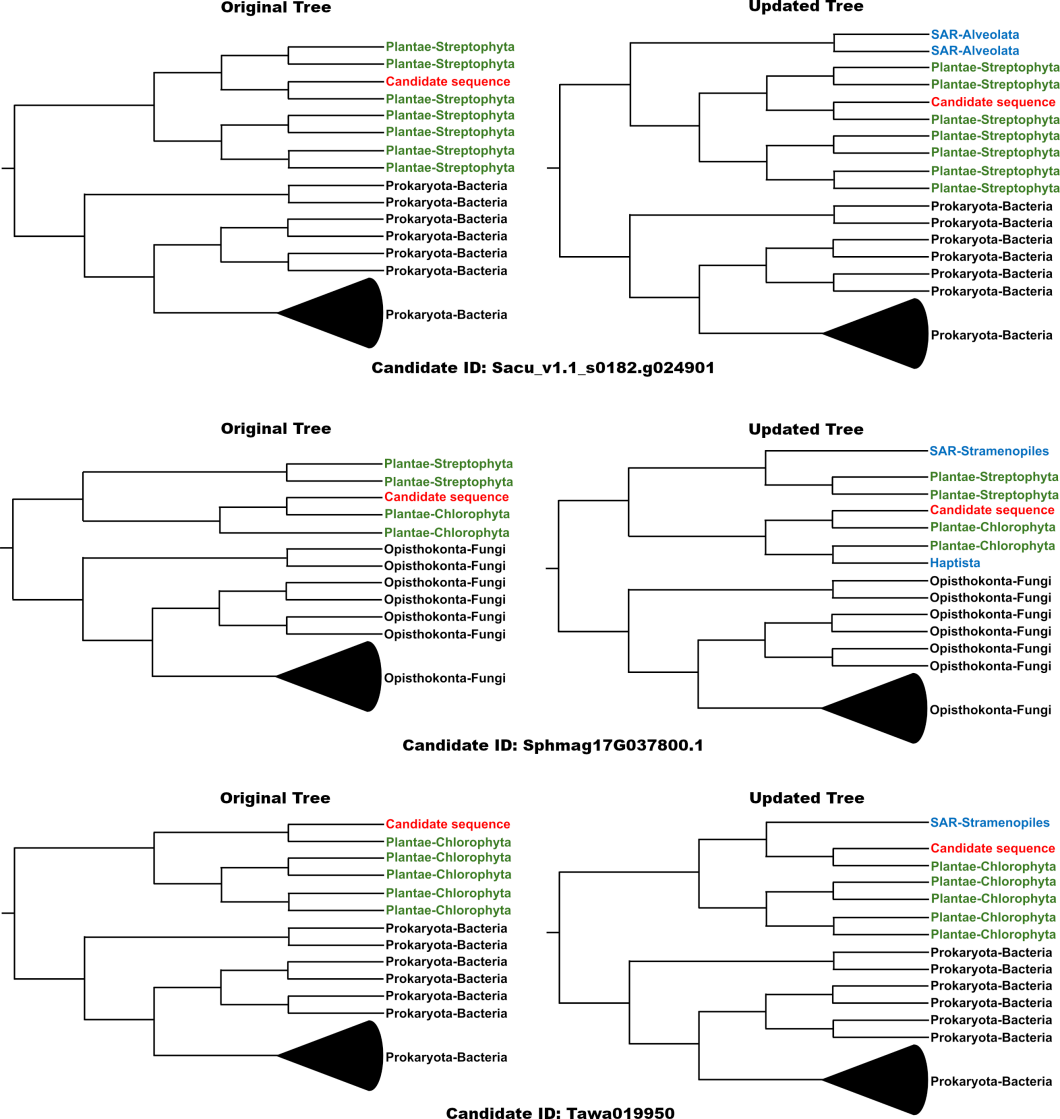
Representative examples of phylogenetic topology shifts following the incorporation of newly identified homologs into updated reconstructions. For each candidate gene, the left panel displays the original phylogeny reported by Wu et al. (2024), while the right panel shows the topology obtained after adding homologs identified through expanded database searches. In the original trees, candidate sequences often cluster in close association with prokaryotic or fungal lineages, a pattern commonly interpreted as evidence supporting interkingdom horizontal gene transfer (iHGT). However, after the inclusion of additional homologs, the revised reconstructions reveal a broader representation of eukaryotic diversity, frequently modifying the inferred evolutionary relationships and diminishing support for iHGT. Candidate sequences are highlighted in red, Plantae sequences in green, sequences included in the original analyses in black, and newly recovered homologs in blue. Collapsed branches encompass multiple sequences from the indicated lineage. Candidate identifiers are provided below each corresponding pair of trees.

In a recent reanalysis of published iHGT candidates in plants, Aguirre-Carvajal and Armijos-Jaramillo (2025a) identified previously unreported homologs both within and outside Plantae. That study showed that only 29.3% of the originally proposed candidates remained consistent with an iHGT interpretation. Among the demoted cases, 188 recovered new homologs that altered the originally inferred topologies associated with iHGT. These additional sequences frequently changed the evolutionary interpretation, in some instances shifting the inferred donor lineage and in others substantially weakening support for an iHGT scenario. For example, Sphmag17G037800.1, originally reported in Wu et al. (2024) as having a fungal origin, was found in our updated analysis to have homologs in SAR and Haptista that cluster monophyletically with the fungal and plant sequences (**Figure 2**). This broader taxonomic distribution prompts a reinterpretation: does this represent multiple independent transfers from fungi, or a gene family that is abundant in fungi but sparsely distributed across other eukaryotic lineages? These results illustrate how rapidly evolutionary inferences can change as new data become available. In some cases, even a one-year interval between homology searches led to markedly different conclusions. More broadly, they highlight the inherent difficulty of reconstructing ancient evolutionary events from single-gene phylogenies and the need for caution in their interpretation (Katz, 2015).

In the same reanalysis of the Wu et al. (2024) dataset, there was identified seven candidates with newly detected homologs within Plantae. Although the presence of these additional sequences does not exclude an HGT origin, it modifies the inferred timing or phylogenetic placement of the putative transfer, underscoring the sensitivity of such conclusions to the data available at the time of analysis. For example, candidate KAI5081312 was originally interpreted as a transfer into Polypodiopsida, but the recovery of a homolog in *Diphasiastrum complanatum* shifts the most parsimonious point of transfer to the Tracheophyta clade. The complete reanalysis performed in Aguirre-Carvajal and Armijos-Jaramillo (2025a) is available at https://zenodo.org/records/17552462.

The critical role of updated homology searches in reassessing HGT candidates has been emphasized in recent studies (Aguirre-Carvajal and Armijos-Jaramillo, 2025b; Aguirre-Carvajal et al., 2025). It is also well recognized that current genomic repositories remain highly incomplete, with a pronounced disparity between the number of described species and those for which genome sequences are available. For example, the Catalogue of Life (Bánki et al., 2025) lists 12,253 Bryophyta species, whereas only 155 genomes were available in NCBI as of 25 November 2025. This gap implies that many homologs remain unsampled, a situation that may substantially bias contemporary estimates of HGT prevalence. Similar discrepancies between taxonomic diversity and genomic representation are evident across most eukaryotic lineages.

## Limitations of current approaches for detecting iHGT

The study by Dupont and Cox (2017) highlights the inherent limitations of existing methods for detecting HGT candidates. In that work, both composition-based approaches and phylogeny-based methods—including gene/species tree reconciliation—were evaluated. For composition-based analyses, the authors found that iHGT cases, even when simulated, were indistinguishable from housekeeping genes with respect to parameters such as %GC content, %GC content at third codon positions, Codon Adaptation Index (CAI), and Effective Number of Codons (ENC). When applying phylogeny-based approaches, particularly reconciliation between species and gene trees, the authors concluded that topological discordances—often interpreted as evidence of HGT—are frequently observed even among housekeeping genes. Consequently, these methods exhibit a high error rate in the identification of HGT events.

One of the most widely used methods for detecting HGT relies on reconstructing the phylogeny of a candidate gene together with its homologs, with the aim of identifying topologies that are more consistent with horizontal rather than vertical inheritance. This explicit phylogenetic approach is often regarded as the gold standard for HGT detection (Keeling and Palmer, 2008). However, the identification of homologs depends critically on the content of the databases queried. Consequently, database choice alone can bias candidate detection and thus constitutes an additional source of instability in the inference of horizontally acquired genes.

A clear example is provided by the comparison between the iHGT candidates reported by Wu et al. (2024) and the same candidates reanalyzed by Aguirre-Carvajal and Armijos-Jaramillo (2025a). Although both studies relied on phylogenetic reconstruction, the reanalysis validated only 25.5% of the original candidates. The principal difference between the two analyses was the reference databases used. In general, smaller databases or those with limited representation of eukaryotic sequences are more likely to yield apparent iHGTs, as previously suggested for fungi by Aguirre-Carvajal et al. (2024).

To test whether this trend also applies to plants, we reanalyzed the Wu et al. (2024) dataset using a modified homolog search strategy. Specifically, we queried only the NCBI NR database (updated November 2024), excluding Phytozome (Goodstein et al., 2012) and Genome Warehouse (Ma et al., 2025). Under these conditions, 32.2% of the original Wu et al. (2024) candidates were validated as potential iHGTs, compared with 25.5% when a more comprehensive database was used in Aguirre-Carvajal and Armijos-Jaramillo (2025a). All data generated in this analysis are provided in **Supplementary Table 1** and at https://zenodo.org/records/17203430

This experiment highlights how sensitive phylogeny-based iHGT detection is to database composition and, more specifically, to the presence or absence of homologs for a given candidate. A similar effect was previously reported in humans by Salzberg et al. (2001). In summary, not only the analytical methods themselves influence the detection of HGT candidates, but the choice of reference database used to identify homologs is a critical determinant. Moreover, when iHGT is inferred from phylogenetic trees, the resulting topologies may be compatible with multiple evolutionary scenarios, and HGT should not be treated as the default explanation.

## Challenges and improvements in iHGT detection methodologies

Given the need to properly assess the real impact of interkingdom HGT in plants, it is necessary to reconsider the methods currently available for evaluating alternative evolutionary hypotheses. Although reconciling gene trees that display apparent iHGT patterns with species trees may seem particularly rigorous, in practice these approaches have limited detection power (Dupont and Cox, 2017), largely because of the wide range of evolutionary trajectories that a given gene may follow. In this context, new strategies have begun to emerge, based on phylogenetic and locus networks reconciled with species trees (Du et al., 2019). In theory, such frameworks could discriminate among a broader set of scenarios, including horizontal transfer, introgression, hybridization, and gene duplication and loss. At the same time, these models attempt to incorporate coalescent processes to identify cases of incomplete lineage sorting. If successful, these approaches could provide a more robust validation of candidate iHGT topologies. Unfortunately, to date, relatively little work has been conducted in this direction.

Beyond the evolutionary hypotheses that can be distinguished using phylogenies of candidate iHGT genes in plants, none of these approaches is sufficient in the absence of adequate homolog representation—particularly from eukaryotes—to reliably reconstruct gene trees. This limitation depends not only on the number of species that have been sequenced, but also on sequencing quality (including the minimization of cross-contamination during genome assembly) and on the accuracy of genome annotation. Third-generation sequencing platforms may help reduce the occurrence of false-positive iHGT candidates in future studies. By generating longer reads and improving assembly continuity, these technologies can minimize the formation of chimeric scaffolds—artificial constructs in which unrelated sequences are erroneously merged during assembly—often arising from cross-contamination or technical artifacts in the sequencing process.

At present, public databases are strongly biased toward prokaryotes—especially bacteria—relative to eukaryotes. In our view, this imbalance largely explains why most reported plant iHGT events appear to originate from bacterial donors. The rationale is straightforward: when reconstructing a phylogeny in which most homologs for a given gene derive from bacteria rather than eukaryotes, the topology is naturally interpreted as horizontal transfer. The problem with this interpretation is that we do not know whether additional eukaryotic homologs exist that could reshape the topology toward a vertical transmission scenario. This is precisely why many previously reported iHGT candidates lose support when they are revisited years later, after new eukaryotic homologs become available. This effect has been repeatedly observed across different organisms (Katz, 2015; Aguirre-Carvajal et al., 2024; Aguirre-Carvajal and Armijos-Jaramillo, 2025b).

It is difficult to estimate how many eukaryotic genomes would need to be sequenced in order to identify plant iHGT candidates with high confidence. Nevertheless, the uncertainty introduced by missing homologs in current databases could, in principle, be quantified. Several alternatives exist. For example, this problem could be modeled using species accumulation curves, as commonly applied in ecology to estimate how many species remain undiscovered in a given ecosystem. A similar framework could be developed to estimate the number of homologs yet to be sequenced. Another possibility would be to transform candidate sequences and their known homologs into embedding space: if a candidate lies in a “diffuse” region or far from the dense clusters formed by eukaryotic protein embeddings, it could be inferred to reside in a zone of high uncertainty. These are only conceptual proposals for a topic that has been scarcely explored, yet for iHGT they may prove essential for estimating the confidence we can place in individual candidates.

## Conclusion

In our view, iHGT should not be treated as the default explanation for unexpected phylogenetic patterns. More parsimonious biological scenarios—together with the substantial uncertainty introduced by missing data—must be carefully evaluated before invoking interkingdom transfer. It is therefore imperative to develop new methodologies for detecting HGT that explicitly account for the evolutionary and methodological biases that can lead to false positives. Without such tools and perspectives, it remains difficult to reliably quantify the true impact that HGT has had on the evolution of plant genomes. Future studies that combine dense eukaryotic sampling with methods capable of discriminating among alternative evolutionary hypotheses will be essential for establishing robust and well-supported cases of plant iHGT.

## Data Availability

The datasets presented in this study can be found in online repositories. The names of the repository/repositories and accession number(s) can be found in the article/**Supplementary Material**. In addition to the data submitted as supplementary material to the journal, please refer to the external data available at https://zenodo.org/records/17203430.
